# Predicting Energy Expenditure During Gradient Walking With a Foot Monitoring Device: Model-Based Approach

**DOI:** 10.2196/12335

**Published:** 2019-10-23

**Authors:** Soon Ho Kim, Jong Won Kim, Jung-Jun Park, Myung Jun Shin, MooYoung Choi

**Affiliations:** 1 Department of Physics and Astronomy and Center for Theoretical Physics Seoul National University Seoul Republic of Korea; 2 Department of Healthcare Information Technology Inje University Gimhae Republic of Korea; 3 Division of Sport Science Pusan National University Busan Republic of Korea; 4 Department of Rehabilitation Medicine Pusan National University Hospital Busan Republic of Korea

**Keywords:** energy expenditure, physical activity, human walking, wearable devices, mHealth

## Abstract

**Background:**

Many recent commercial devices aim at providing a practical way to measure energy expenditure. However, those devices are limited in accuracy.

**Objective:**

This study aimed to build a model of energy consumption during walking applicable to a range of sloped surfaces, used in conjunction with a simple, wearable device.

**Methods:**

We constructed a model of energy consumption during gradient walking by using arguments based in mechanics. We built a foot monitoring system that used pressure sensors on the foot insoles. We did experiments in which participants walked on a treadmill wearing the foot monitoring system, and indirect calorimetry was used for validation. We found the parameters of the model by fitting to the data.

**Results:**

When walking at 1.5 m/s, we found that the model predicted a calorie consumption rate of 5.54 kcal/min for a woman with average height and weight and 6.89 kcal/min for an average man. With the obtained parameters, the model predicted the data with a root-mean-square deviation of 0.96 kcal/min and median percent error of 12.4%.

**Conclusions:**

Our model was found to be an accurate predictor of energy consumption when walking on a range of slopes. The model uses few variables; thus, it can be used in conjunction with a convenient wearable device.

## Introduction

Physical inactivity, despite its well-known health risks [1,2], continues to be a serious public health issue [3]. Recently, various wearable devices, including wristbands and mobile phones, have offered a way to track physical activity throughout the day. Such devices can be used in ambulatory conditions by individuals or in clinical settings to monitor patients’ physical activity.

Many of these devices use an accelerometer-based method to predict energy expenditure [4-6]. However, these methods are limited in precision [7]. A basic, common assumption used is that the calorie consumption rate is proportional to the walking velocity. A GPS tracker can then be used to measure the walking distance and then compute the total energy consumption. However, this method is limited in accuracy and may not be feasible indoors.

### Literature Review

The energetics of human locomotion has been closely studied for decades. Early studies focused on energy expenditure during walking [8-12] and running [13-16], and made comparisons with the energy expenditures of other animals [17]. Most relevantly, studies on walking energetics found a proportional relationship between energy expenditure and the square of the velocity. These early studies showed that reasonable accuracy can be attained with simple relations, despite the complexity of the act of walking. More recently, detailed models of walking dynamics have been presented that examine more closely the mechanics of walking [18-24]. These biomechanical models aim to explain human gait patterns via energy minimization. Also studied have been movements of the arm [25,26] and the head and trunk [27], as well as gait patterns in special groups of interest [28,29]. Such models have also been used in the field of robotics in developing walking robots [30].

Previous studies were primarily of academic interest, although inexpensive commercial devices have recently been made available for personal or clinical use. Such devices offer noninvasive ways to measure daily caloric consumption, and they have been assessed by numerous validation studies in the literature [31-36]. The most common types of commercially available products include the wrist-worn accelerometer and devices based on heart rate monitors. Although these devices are good predictors of the number of steps and heart rate, accurate prediction of energy expenditure is yet to be achieved [37]. These validation studies test for various settings; however, they usually lack a discussion of the model or algorithm used in their predictions.

This study proposes a model of walking energetics applicable to a range of slopes. The model is based on a simple equation and uses data from a wearable device. The method uses a foot monitoring system that can sense footsteps, which allows for direct measurement of step frequency. We found that a high-accuracy model can be developed for a range of upward and downward slopes. The fact that it is based on a direct measurement of footsteps allows the device to be versatile and applicable to diverse walking situations. The ability to track expenditure while walking on sloped surfaces is helpful for sloped outdoor ground and also indoor use of stairs or sloped treadmills.

## Methods

### Experimental Procedure

For model development and validation, an experiment was devised in which 73 healthy participants (34 female, 39 male) walked on a treadmill. The participants had a mean age of 43.6 (SD 15.0) years, mean height of 168.3 (SD 10.5) cm, and mean weight of 68.1 (SD 12.1) kg. Participants were selected from healthy volunteers (age 20 to 60 years) who registered in the department of Sport Science, Pusan National University, Busan, Korea. We excluded participants who had cardiovascular, musculoskeletal, or neurological disorders to avoid any confounding factors or biases. The participants were asked to walk on a treadmill at various values of the incline angle, *Theta,* and speed, *v.* Specifically, the angle was taken to be 0° (indicating no incline); 4°, 9°, and 14° (uphill); and −4°, −9°, and −14° (downhill). It was observed that calorie consumption took approximately 30 seconds to stabilize to a linear rate while walking. Each walking measurement lasted approximately 5 minutes to ensure a sufficiently long sample.

Calorie consumption was measured with a COSMED K4b2 portable gas analyzer system. This indirect calorimetry, based on the gas analyzer system, measures oxygen consumption, from which energy expenditure is computed. This method has been validated as an accurate measure through numerous comparative studies [38-40] and is used as a criterion measure in many validation studies [31-33,35-37]. The gas analyzer was worn during the treadmill experiment, and it recorded a time series of cumulative calorie consumption. To eliminate noise associated with the beginning and end of the experiment, we discarded data for the first 50 seconds and the final 10 seconds before computing the energy consumption rate. Then the basal metabolic rate [41] was subtracted to obtain the energy expenditure associated with walking, which is denoted by *P.*

**Figure 1:**
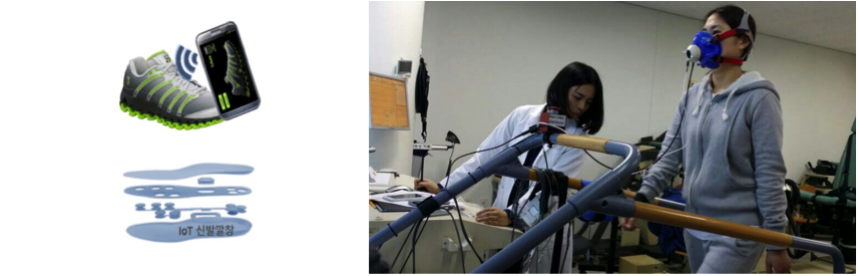
Illustration of the foot monitoring system (left) and a picture of a participant walking on an uphill treadmill wearing the K4b2 portable gas analyzer (right).

Each participant also wore a foot monitoring system, consisting of shoe insoles equipped with eight pressure sensors. The insole used was a prototype developed by 3L Labs (Seoul, Korea), and provided to us for research purposes. A depiction of the foot monitoring system and the experimental setup is given in Figure 1. A Fitbit Surge, a wrist-worn accelerometer device, was also worn by each participant to compare the accuracy of its caloric consumption prediction. This study was approved by the Institutional Review Board of Pusan National University, Busan, Korea. All participants provided written informed consent (PNU IRB/2015_33_HR).

**Figure 2:**
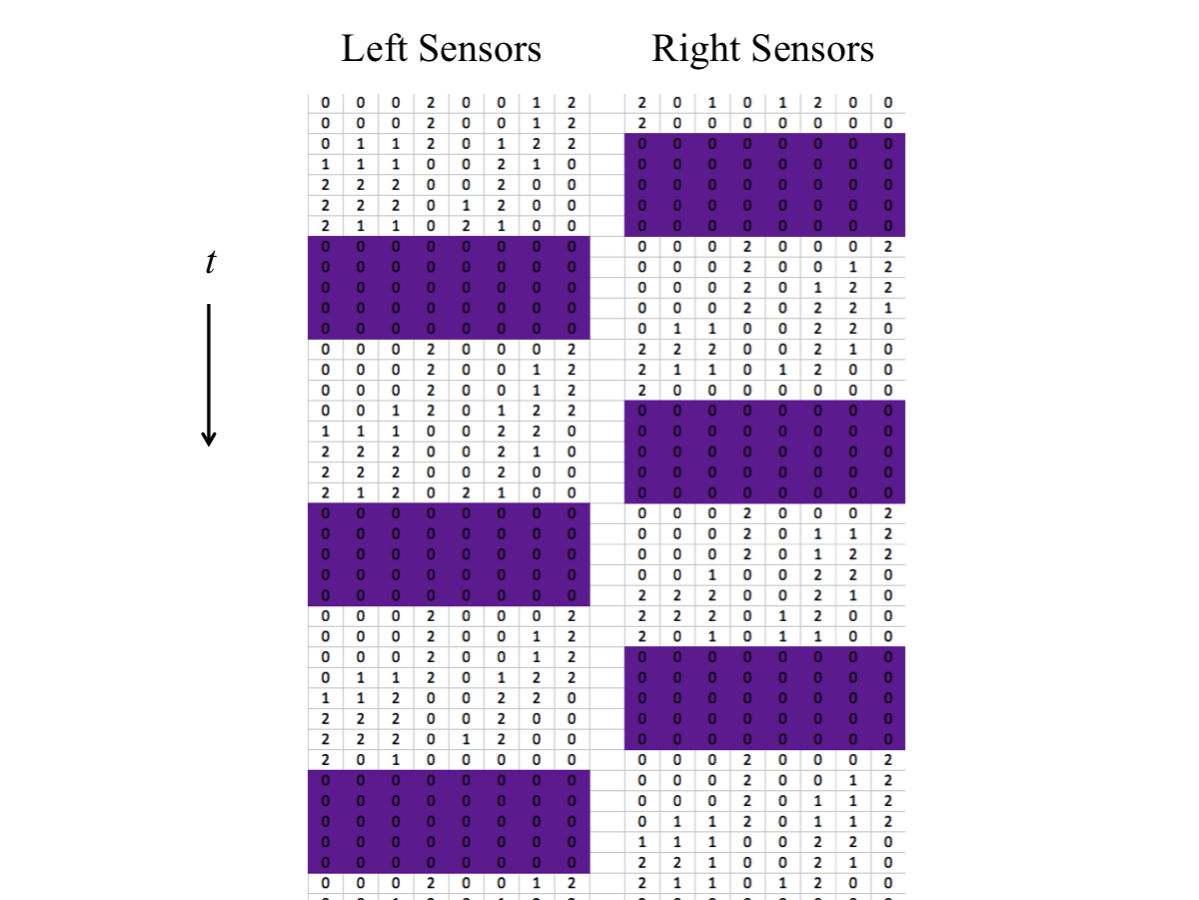
A sample of 4 seconds of raw data from the pressure sensors. The vertical position of each number of the array indicates the time, ordered from top to bottom at an increment of 0.1 seconds. Each column denotes a sensor, with left foot and right foot separated. The colored portions indicate when our algorithm decided the foot was off the ground.

**Figure 3:**
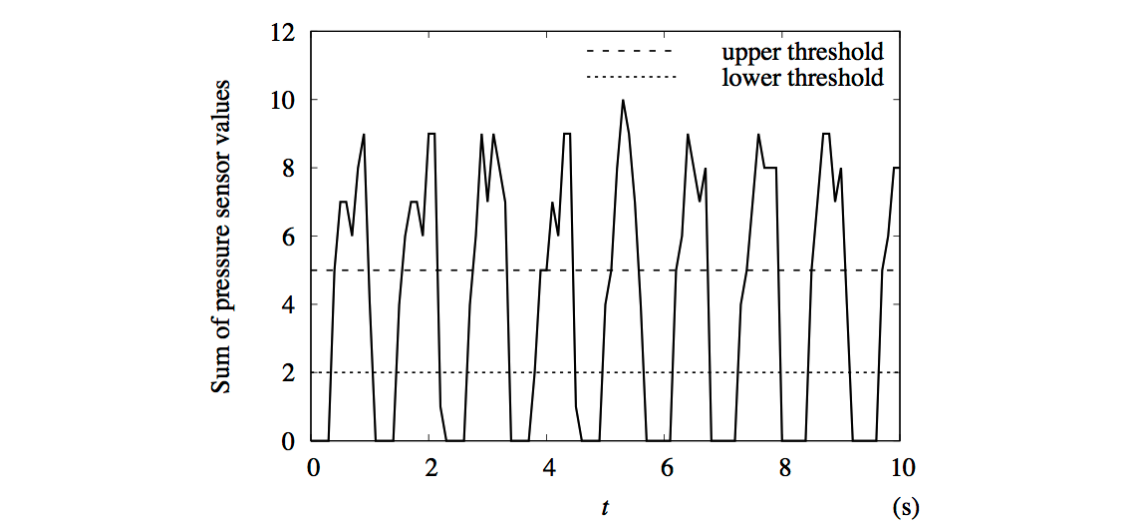
Graph of total pressure from the left foot sole over an interval of 10 seconds obtained from the foot monitoring system from the same data as presented in Figure 2. The two dashed lines indicate the upper and lower thresholds used to calculate the step frequency.

A value of 0, 1, or 2 indicated the pressure on each of the pressure sensors and was recorded with a frequency of 10 Hz, which resulted in an array of 16 integers for each time step of 0.1 s. A snippet from example data is shown in Figure 2. From the pressure sensor data, we were able to extract the step frequency, *f.* We performed this by examining the sum of the pressure sensor values at each time step. An example is shown in Figure 3. Although it is natural to consider the foot to be off the ground when this sum is 0, this can result in erroneous results if one or more of the pressure sensors remain at a value above 0 throughout the entire step cycle, either due to a faulty sensor or residual pressure. We found that better accuracy was achieved when high and low thresholds were used. This was done by first assigning the on-ground status to the first-time step, and then sequentially assigning either the on-ground or off-ground status to each following time step. If the previous time step was on-ground and the pressure sum was below the lower threshold, we assigned the off-ground status to that time step; if the threshold was not crossed, the time step was left in on-ground status. If the previous status was off-ground, the on-ground status was assigned if the pressure sum was above the upper threshold, and the off-ground status was assigned otherwise. Threshold values between 1 and 10 were tested and compared with manually assigned steps. Lower and upper threshold values of 2 and 5, shown in Figure 3, were found to produce accurate results.

After assigning a status to each time step, we counted the number of transitions from the on-ground to off-ground status and divided it by the time interval to obtain the frequency. As with the gas analyzer data, we omitted data for the first 50 s and the final 10 s. Only one shoe insole is required to calculate the step frequency; however, we used the average of both sides in this study.

### Model

**Figure 4:**
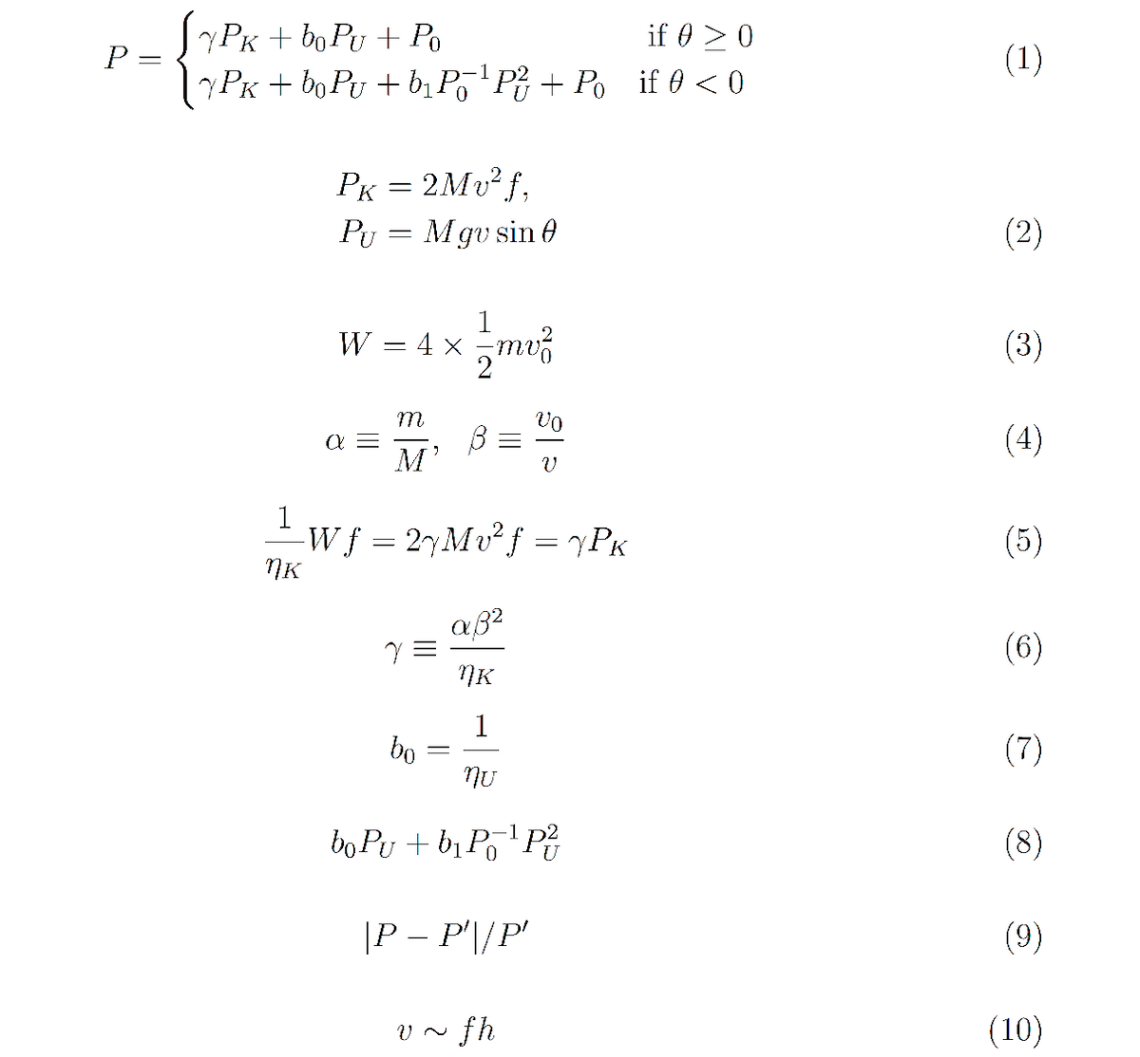
List of equations of the model of energy expenditure during walking.

Our model was constructed from considering the energy changes involved in walking. Suppose a participant with body mass *Mu* is walking with average speed *Nu* on a surface inclined by *Theta* from the horizontal. The participant is swinging their legs with frequency *f.* The energy consumption rate, *Rho,* is given by equation 1 (Figure 4). Here positive and negative values of the slope, *Theta,* of the walking surface correspond to walking uphill and downhill, respectively. *Rho*_K_ and *Rho*_U_ are rates of changes in the kinetic energy and in the potential energy, respectively, whereas coefficients *Gamma,* *b*_Tau_, *b*_1_, and *Rho*_0_ are parameters to be determined empirically from the data. The energy change rates for *Rho*_K_ and *Rho*_U_ are given in equation 2 (Figure 4). In the following, we give an explanation of each term in consideration of energy.

#### Kinetic Energy Component

We first consider walking on a horizontal surface (ie, *Theta*=0). When walking on a treadmill, the upper body moves in a relatively constant velocity, with the moving legs supporting this movement. The legs swing back and forth relative to the upper body’s position, undergoing an acceleration-deceleration cycle. We postulated that the energy expenditure was proportional to the kinetic energy change of the legs. The work done on the legs during each walking cycle is given by equation 3 (Figure 4). Here *m* is the mass of each leg, *v*_0_ is the maximum speed of each leg’s center of mass, and the factor of 4 accounts for the two legs each undergoing acceleration and then deceleration. This differs from the assumption that the legs swing like a pendulum, in which case gravity would do the work.

Since we usually have no way to easily measure leg mass or leg velocity, we defined two ratios: (1) the ratio *α* of the leg mass, *m,* to the body mass, *M;* and (2) the ratio *β* of the maximum velocity, *v*_0_, of the leg to the average walking speed, *v* (equation 4 in Figure 4).

This allowed us to rewrite equation 3 as 
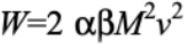
, giving an expression for the work done per cycle. Assuming that the human body converts chemical energy into kinetic energy with efficiency *η_K_,* the energy consumption rate due to the kinetic energy is given by equation 5 (Figure 4). In writing the right-hand side of equation 5, the measurable terms are grouped into *P_K_* as in equation 2, whereas the rest are grouped into dimensionless coefficient *γ,* given by equation 6.

#### Potential Energy Component

When walking on a horizontal surface perpendicular to the direction of gravity, there is no net change in potential energy. It changes when the subject is walking up or down a slope. We first considered upward inclines. When one walks up a slope of angle *Theta* at speed *v* parallel to the surface, their potential energy, *U,* changes at a rate *dU*/*dt*=*P_U_,* given by equation 2 (Figure 4). For simplicity, we further assumed that when walking up a slope, additional energy proportional to this term is required. Accordingly, the energy expenditure rate associated with the changing potential energy is given by *b*_0_ *P_U_,* where *b*_0_ is the inverse of the efficiency, *η_U_,* (equation 7 in Figure 4) with which the body converts stored energy to potential energy.

One might consider simply using the same formula for downhill inclines, in which case the term *b*_0_ *P_U_*=*b*_0_ *Mgv* sin*Theta* becomes negative. This would imply that when walking downslope, the change in potential energy can be converted into kinetic energy, thereby subtracting from the total energy cost. However, this leads to a nonsensical result for higher slopes, as it can lead to negative energy consumption. When a downhill slope is steeper than a certain angle, the subject would need to exert a frictional force to prevent from falling forward or walking too fast. Therefore, *b*_0_ *P_U_* does not provide an adequate description of the energy expenditure in this case.

**Figure 5:**
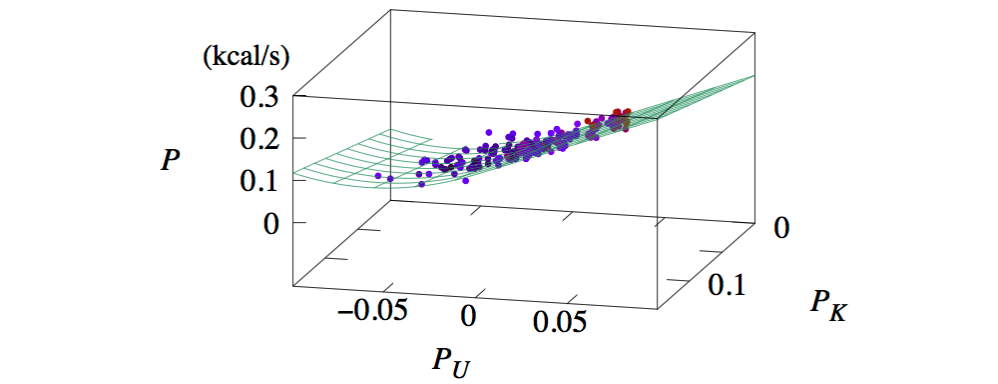
Three-dimensional scatterplot of data (dots) and model prediction (lines) of P versus P_U_ and P_K_ for women.

**Figure 6:**
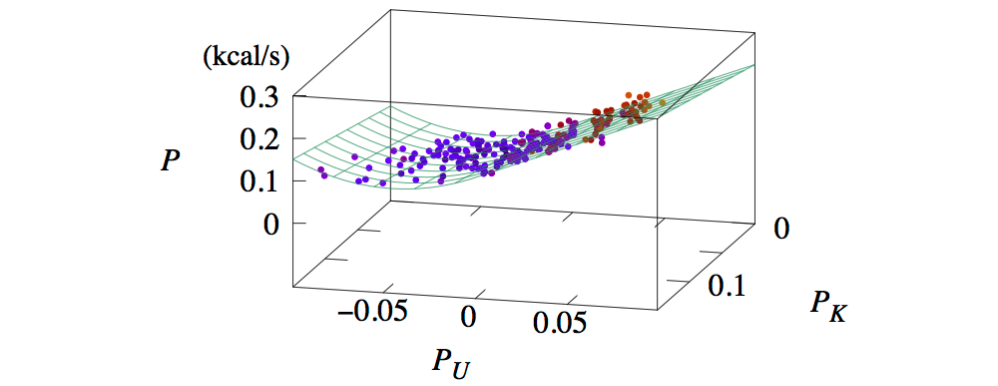
Three-dimensional scatterplot of data (dots) and model prediction (lines) of P versus PU and PK for men.

Figure 5 and 6 present scatterplots of the data in the three-dimensional space (*P_K_,P_U_,P*) for women and men, respectively. This visualization shows that *P* first decreases then increases as *P_U_* is decreased from zero. Such a parabolic shape indicates the presence of a quadratic term; thus, we added to *P* a term proportional to *P_U_*^2^. The energy expenditure associated with potential energy in the case of downhill walking is given by equation 8 (Figure 4). The second term is multiplied by *P*_0_^-1^ so that the coefficient *b*_1_ is kept dimensionless. In other words, *b*_1_ is the coefficient of the quadratic term in the case of downhill walking in units of *P*_0_. This leads to the full model, described by equation 1 (Figure 4).

### Linear Regression

The preceding model described leaves parameters *ϒ,* *b*_0_, *b*_1_, and *P*_0_ to be determined. We obtained these parameters by first taking data for flat and uphill surfaces (*Theta*≥0) and performing multiple linear regression through the use of the first equation in equation 1 (Figure 4) with *Υ,* *b*_0_, and *P*_0_ as fitting parameters. The adjusted *R*^2^ value for the fits of both women and men was .83. Then *b*_1_ was obtained via fitting the second equation of equation 1 (Figure 4) to flat and downslope data (*Theta*≤0). During this secondary fit, *ϒ,* *b*_0_, and *P*_0_ were set constant at the values obtained earlier.

## Results

The full set of coefficients, obtained through linear regression, is given in Table 1. The dependency of *P* on *P_K_* and *P_U_* is represented by the surfaces in Figures 5 and 6 . Due to the piecewise functional form of the model (equation 1 in Figure 4), the prediction plane has no curvature for *P_U_*>0 but does in the region *P_U_*<0.

**Table 1:** Coefficients for the full model reported with the root-mean-square deviation (RMSD) on comparison with data. The values were obtained by two linear regressions.

Coefficient	Units	Women	Men
*γ*	—	0.662	0.517
*b*_0_	—	1.591	1.694
*b*_1_	—	0.575	1.086
*P*_0_	kcal/s	0.042	0.058
RMSD	kcal/s (kcal/min)	0.016 (0.96)	0.016 (0.96)

**Figure 7:**
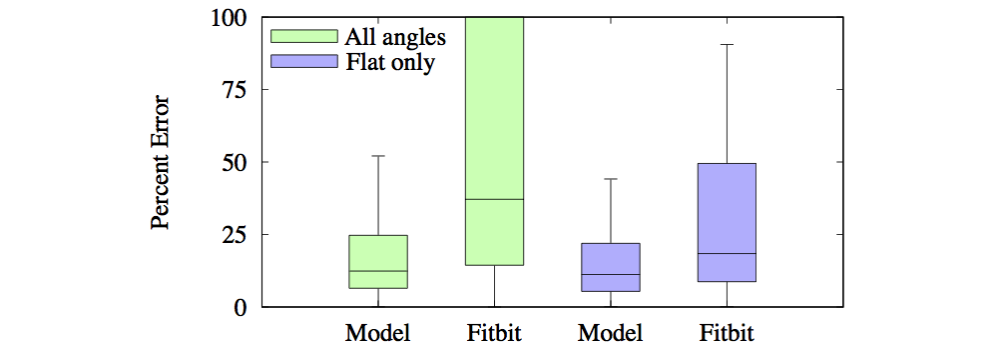
Boxplots of the percent errors of predictions made by the model and Fitbit Surge. Errors have been estimated via equation 9 in Figure 4.

The fit resulted in a root-mean-square deviation (RMSD) of 0.96 kcal/min for both women and men. A boxplot of the percentage errors of all trials is given in Figure 7, in which the errors have been calculated according to equation 9 in Figure 4.

Here *P* is the prediction by the method whereas *P'* is the standard given by the gas analyzer. The median errors were 16.9% for women, 11.2% for men, and 12.4% for both groups. These errors are substantially lower than those found in a validation study for multiple commercial devices, which yielded median accuracies of 28.6% to 35.0% across devices for walking [37].

The predictions made by Fitbit Surge had an RMSD of 2.58 kcal/min (2.7 times that of the model) and a median percent error of 37.3% (3 times that of the model). However, this high error was mostly due to inaccuracies in sloped walking. When restricted to flat surfaces, the Fitbit Surge’s accuracy increased dramatically, whereas the model’s accuracy increased moderately. The Fitbit Surge’s RMSD on flat surfaces was 1.82 kcal/min (2.3 times that of the model, 0.79 kcal/min), and the median percent error was 18.4% (1.6 times that of the model, 11.2%). Distributions of percent errors are portrayed with boxplots in Figure 7.

**Figure 8:**
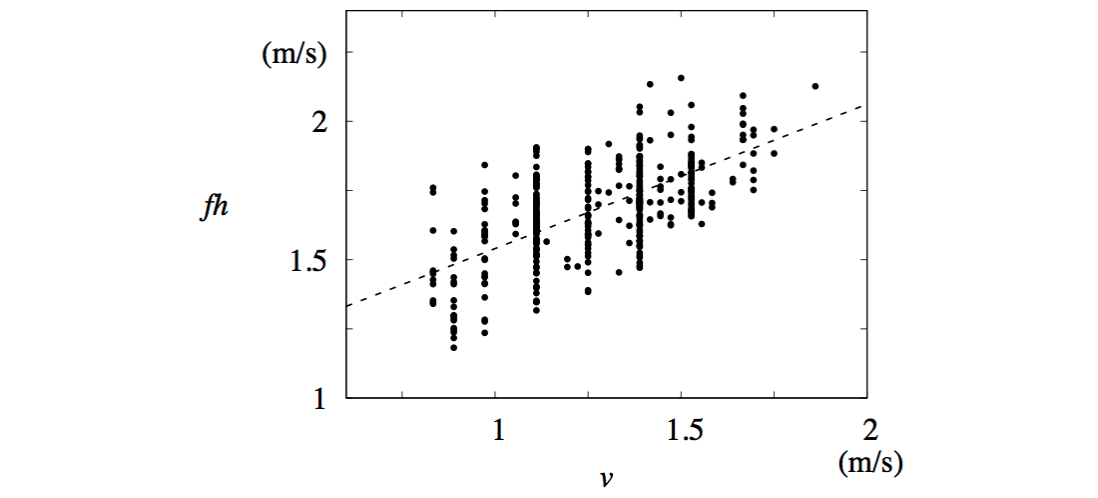
Step frequency, *f*, multiplied by height, *h*, plotted against average walking speed, *v*. Least squares fit line fh = 0.52v+1.02 (m/s) is also shown.

Before discussing the implications of these results, we note that the variables *v* and *f* are not independent. If *l* is the average length of a step, then *v* = *f l.* Assuming the approximate relation *h* ≈ *l,* where *h* is the subject’s height, we obtain *v* ~ *fh* (equation 10 in Figure 4). This relation was observed in the data, as shown in Figure 8.

Equation 7 implies that *η_U_*=0.547 for women and 0.596 for men. In principle, *ϒ* depends on *α,* *β,* and *η_K_.* We assumed the average value of *α*=0.185 for women and 0.165 for men, obtained from an anatomical reference [42], and that *η_K_*=*η_U_.* Taking these values and the fitting result for *ϒ,* we obtained from equation 6 (Figure 4) the ratio *β* with values 1.47 for women and 1.36 for men. This difference in the average may reflect the difference in the average height between women and men. Specifically, equations 4 and 10 (Figure 4) imply *β*=*v*_0_/*v* ~ *v*_0_/*f h.* The ratio of the value of *β* for women to that for men equaled 1.08, whereas the ratio of the average height of men to that of women equaled 1.11.

## Discussion

### Principal Results

We developed a model based on rates of change in kinetic and potential energies. In general, it predicts linear dependence of the energy consumption on these rates; in particular, it predicts quadratic dependence of the energy consumption on the potential energy change in the case of downhill walking. The method, used in conjunction with a foot monitoring system, predicts energy expenditure with an RMSD of 0.98 kcal/min and a median percent error of 12.4%, lower than those of wrist-worn commercial devices in predicting energy expenditure for walking. With one simple piecewise function, the model adequately predicts energy expenditure for walking in a wide range of the gradient.

Notice the differences in parameter values between women and men. The appreciable difference in the value of *b*_1_ between men and women may result from the difference in walking posture; this is beyond the scope of this work and left for future study. In principle, the parameters are fit for each individual and should vary by subject. Thus, Table 1 presents average values of the coefficients within each gender. Even so, it is remarkable that a high degree of accuracy is observed.

### Limitations

Although the model accounts for varying body mass and step frequency (cadence), this does not account for additional individual variations in parameter values due to walking gait and body dimensions. There may be ways to account for such variations without complicating the model. In addition, because the treadmill incline lies between 14° uphill and 14° downhill, we are not able to validate the model for more extreme slopes [43]. In addition, the method has not been tested and calibrated for outdoor walking or variable temperatures and altitudes. However, we believe that our pilot study provides a groundwork for follow-up studies under more ambulatory conditions.

### Comparison With Prior Work

Prior studies have noted the strong correlations between *P* and *v*^2^ for level walking [10]. The authors have also similarly considered additional energy expenditure when walking uphill, attributing it to vertical lift work. In contrast, our study proposes a simple formula that predicts energy consumption reasonably well for horizontal, uphill, and downhill surfaces within a unified framework. In addition, Cotes and Meade [9] made use of individual measurements, including resting metabolic rate and leg length. Our model shows that high accuracy can be achieved via reasonable assumptions used in conjunction with a wearable, mobile device.

Other existing studies have studied energy expenditure during uphill and downhill walking [43,44]. The authors reported a minimum energy cost when walking 10° downhill, which is consistent with our results. These studies did not incorporate varying walking speed and body weight, and relied on regression analysis with those variables kept constant. Our study offers a simple formula that applies to various walking speeds and subjects, while also accounting for the surface gradient.

Our method fits separately for women and men. Prior validation studies have found differences in the accuracy of devices between the two genders. A comparative validation study found that gender was one of the strongest predictors for accuracy, with a rate significantly higher for men than for women [37]. Our results suggest that similar error rates for both genders can be achieved.

### Conclusions

We have developed a model that predicts energy expenditure during walking on a gradient surface between 14° uphill and 14° downhill, with an RMSD of 0.98 kcal/min. The model has been used in conjunction with a wearable device, the foot monitoring system, which directly measures footsteps. Thus, it offers an accessible method of measuring energy expenditure in realistic walking settings, where gradient walking is common. Future work may test equation 1 (Figure 4) in a wider range of values in the *P_K_*−*P_U_* space. Testing the method on outdoor walking is also desirable for further validation. Although not yet explored, the device could also be used in conjunction with other activity monitoring devices, such as wrist-worn ones, to produce more accurate measures of energy expenditure.

## Abbreviations

RMSD: root-mean-square deviation

## References

[1] Nocon M, Hiemann T, Müller-Riemenschneider F, Thalau F, Roll S, Willich SN (2008). Association of physical activity with all-cause and cardiovascular mortality: a systematic review and meta-analysis. Eur J Cardiovasc Prev Rehabil.

[2] Wen CP, Wai JP, Tsai MK, Yang YC, Cheng TY, Lee M, Chan HT, Tsao CK, Tsai SP, Wu X (2011). Minimum amount of physical activity for reduced mortality and extended life expectancy: a prospective cohort study. Lancet.

[3] Trost SG, Blair SN, Khan KM (2014). Physical inactivity remains the greatest public health problem of the 21st century: evidence, improved methods and solutions using the ‘7 investments that work’ as a framework. Br J Sports Med.

[4] Maddison R, Gemming L, Monedero J, Bolger L, Belton S, Issartel J, Marsh S, Direito A, Solenhill M, Zhao J, Exeter DJ, Vathsangam H, Rawstorn JC (2017). Quantifying human movement using the Movn smartphone app: validation and field study. JMIR Mhealth Uhealth.

[5] Montoye HJ, Washburn R, Servais S, Ertl A, Webster JG, Nagle FJ (1983). Estimation of energy expenditure by a portable accelerometer. Med Sci Sports Exerc.

[6] Meijer GA, Westerterp KR, Koper H, ten Hoor F (1989). Assessment of energy expenditure by recording heart rate and body acceleration. Med Sci Sports Exerc.

[7] Chen KY, Sun M (1997). Improving energy expenditure estimation by using a triaxial accelerometer. J Appl Physiol (1985).

[8] Passmore R, Durnin JV (1955). Human energy expenditure. Physiol Rev.

[9] Cotes JE, Meade F (1960). The energy expenditure and mechanical energy demand in walking. Ergonomics.

[10] Dill DB (1965). Oxygen used in horizontal and grade walking and running on the treadmill. J Appl Physiol.

[11] Menier DR, Pugh LG (1968). The relation of oxygen intake and velocity of walking and running, in competition walkers. J Physiol.

[12] van der Walt WH, Wyndham CH (1973). An equation for prediction of energy expenditure of walking and running. J Appl Physiol.

[13] Holt KG, Hamill J, Andres RO (1991). Predicting the minimal energy costs of human walking. Med Sci Sports Exerc.

[14] Margaria R, Cerretelli P, Aghemo P, Sassi G (1963). Energy cost of running. J Appl Physiol.

[15] Zarrugh MY, Radcliffe CW (1978). Predicting metabolic cost of level walking. Europ J Appl Physiol.

[16] Cavanagh PR, Williams KR (1982). The effect of stride length variation on oxygen uptake during distance running. Med Sci Sports Exerc.

[17] Steudel K (1996). Limb morphology, bipedal gait, and the energetics of hominid locomotion. Am J Phys Anthropol.

[18] Minetti A, Alexander R (1997). A theory of metabolic costs for bipedal gaits. J Theor Biol.

[19] Alexander RM (1992). A model of bipedal locomotion on compliant legs. Philos Trans R Soc Lond B Biol Sci.

[20] Doke J, Donelan JM, Kuo AD (2005). Mechanics and energetics of swinging the human leg. J Exp Biol.

[21] Kuo A (2002). Energetics of actively powered locomotion using the simplest walking model. J Biomech Eng.

[22] Kuo AD, Donelan JM, Ruina A (2005). Energetic consequences of walking like an inverted pendulum: step-to-step transitions. Exerc Sport Sci Rev.

[23] Ruina A, Bertram JE, Srinivasan M (2005). A collisional model of the energetic cost of support work qualitatively explains leg sequencing in walking and galloping, pseudo-elastic leg behavior in running and the walk-to-run transition. J Theor Biol.

[24] Osaki Y, Kunin M, Cohen B, Raphan T (2006). Three-dimensional kinematics and dynamics of the foot during walking: a model of central control mechanisms. Exp Brain Res.

[25] Donker SF, Mulder T, Nienhuis B, Duysens J (2002). Adaptations in arm movements for added mass to wrist or ankle during walking. Exp Brain Res.

[26] Zehr EP, Haridas C (2003). Modulation of cutaneous reflexes in arm muscles during walking: further evidence of similar control mechanisms for rhythmic human arm and leg movements. Exp Brain Res.

[27] Hirasaki E, Moore ST, Raphan T, Cohen B (1999). Effects of walking velocity on vertical head and body movements during locomotion. Exp Brain Res.

[28] Hausdorff JM, Yogev G, Springer S, Simon ES, Giladi N (2005). Walking is more like catching than tapping: gait in the elderly as a complex cognitive task. Exp Brain Res.

[29] Blin O, Ferrandez A, Serratrice G (1990). Quantitative analysis of gait in Parkinson patients: increased variability of stride length. J Neurol Sci.

[30] Collins SH, Wisse M, Ruina A (2016). A Three-Dimensional Passive-Dynamic Walking Robot with Two Legs and Knees. Int J Robotics Res.

[31] Adam Noah J, Spierer DK, Gu J, Bronner S (2013). Comparison of steps and energy expenditure assessment in adults of Fitbit Tracker and Ultra to the Actical and indirect calorimetry. J Med Eng Tech.

[32] Brugniaux JV, Niva A, Pulkkinen I, Laukkanen RM, Richalet J, Pichon AP (2008). Polar Activity Watch 200: a new device to accurately assess energy expenditure. Br J Sports Med.

[33] Härtel S, Gnam J, Löffler S, Bös K (2010). Estimation of energy expenditure using accelerometers and activity-based energy models—validation of a new device. Eur Rev Aging Phys Act.

[34] Takacs J, Pollock CL, Guenther JR, Bahar M, Napier C, Hunt MA (2014). Validation of the Fitbit One activity monitor device during treadmill walking. J Sci Med Sport.

[35] Diaz KM, Krupka DJ, Chang MJ, Peacock J, Ma Y, Goldsmith J, Schwartz JE, Davidson KW (2015). Fitbit®: An accurate and reliable device for wireless physical activity tracking. Int J Cariol.

[36] Tucker WJ, Bhammar DM, Sawyer BJ, Buman MP, Gaesser GA (2015). Validity and reliability of Nike + Fuelband for estimating physical activity energy expenditure. BMC Sports Sci Med Rehabil.

[37] Shcherbina A, Mattsson C, Waggott D, Salisbury H, Christle J, Hastie T, Wheeler M, Ashley E (2017). Accuracy in Wrist-Worn, Sensor-Based Measurements of Heart Rate and Energy Expenditure in a Diverse Cohort. J Pers Med.

[38] Ainslie PN, Reilly T, Westerterp KR (2003). Estimating human energy expenditure. Sports Med.

[39] Levine JA (2007). Measurement of energy expenditure. Public Health Nutr.

[40] Haugen HA, Chan L, Li F (2017). Indirect calorimetry: a practical guide for clinicians. Nutr Clin Pract.

[41] Mifflin MD, St JS, Hill LA, Scott BJ, Daugherty SA, Koh YO (1990). A new predictive equation for resting energy expenditure in healthy individuals. Am J Clin Nutr.

[42] Plagenhoef S, Evans FG, Abdelnour T (1983). Anatomical data for analyzing human motion. Res Q Exerc Sport.

[43] Minetti AE, Moia C, Roi GS, Susta D, Ferretti G (2002). Energy cost of walking and running at extreme uphill and downhill slopes. J Appl Physiol.

[44] Minetti AE, Ardigo LP, Saibene F (1993). Mechanical determinants of gradient walking energetics in man. J Physiol.

